# Advancements and applications of single-cell multi-omics techniques in cancer research: Unveiling heterogeneity and paving the way for precision therapeutics

**DOI:** 10.1016/j.bbrep.2023.101589

**Published:** 2023-11-29

**Authors:** Anqi Liang, Ying Kong, Zhihong Chen, Yishu Qiu, Yanhong Wu, Xiao Zhu, Zesong Li

**Affiliations:** a Computational Systems Biology Lab (CSBL), The Marine Biomedical Research Institute, Guangdong Medical University, Zhanjiang, China; b Department of Clinical Laboratory, The Third People's Hospital of Hubei Province, Wuhan, China; c Department of Biology, College of Arts and Science, New York University, New York, USA; d Guangdong Provincial Key Laboratory of Systems Biology and Synthetic Biology for Urogenital Tumors, Shenzhen Key Laboratory of Genitourinary Tumor, Department of Urology, The First Affiliated Hospital of Shenzhen University, Shenzhen Second People's Hospital (Shenzhen Institute of Translational Medicine), Shenzhen, China

**Keywords:** Cancer therapeutics, Precision on-omics, Single-cell multi-omics techniques, Live cell imaging, Protein detection

## Abstract

Single-cell multi-omics technologies have revolutionized cancer research by allowing us to examine individual cells at a molecular level. Unlike traditional bulk omics approaches, which analyze populations of cells together, single-cell multi-omics enables us to uncover the heterogeneity within tumors and understand the unique molecular characteristics of different cell populations. By doing so, we can identify rare subpopulations of cells that are influential in tumor growth, metastasis, and resistance to therapy. Moreover, single-cell multi-omics analysis provides valuable insights into the immune response triggered by various therapeutic interventions, such as immune checkpoint blockade, chemotherapy, and cell therapy. It also helps us better understand the intricate tumor microenvironment and its impact on patient prognosis and response to treatment. This comprehensive review focuses on the recent advancements in single-cell multi-omics methodologies, with an emphasis on single-cell multi-omics technologies. It highlights the important role of these techniques in uncovering the complexity of tumorigenesis and its multiple applications in cancer research, as well as their equally great contributions in other areas such as immunology. Through single-cell multi-omics, we gain a deeper understanding of cancer biology and pave the way for more precise and effective therapeutic strategies. Apart from those above, this paper also aims to introduce the advancements in live cell imaging technology, the latest developments in protein detection techniques, and explore their seamless integration with single-cell multi-omics technology.

## Highlights

• Single-cell multi-omics reveals intricate cellular traits, surpassing bulk analysis limits.
• Single-cell multi-omics dissects tumor heterogeneity, informs precision targets.
• Personalized therapies driven by patient-specific biomarkers for diverse cancer types.
• Unveils tumor microenvironment, advancing disease insights, diagnostics & therapies.

## Abbreviations

DNA: deoxyribonucleic acid
RNA: ribonucleic acid
Smart-Seq: switching mechanism at 5′ end of the RNA transcript
CEL-Seq: cell expression by linear amplification and sequencing
MM: Multiple myeloma
PPIA: peptidyl-prolyl isomerase A
MPNs: myeloproliferative neoplasms
HL, Hodgkin lymphoma
DLBCL, diffuse large B-cell lymphoma
HBV: hepatitis B virus
MCL, mantle cell lymphoma
FL, follicular lymphoma
NHC: nonhematopoietic cell
CRC: colorectal carcinoma
CRLM: colorectal cancer liver metastases
BC: bladder cancer
EC: esophageal cancer
ESCC: esophageal squamous cell carcinoma
EAC: esophageal adenocarcinoma
TKI: tyrosine kinase inhibitor
EGFR-TKI: epidermal growth factor receptor tyrosine kinase inhibitor
PC: prostate cancer
RCC: renal cell carcinoma
WNT: wingless/integrated

## 1 Introduction

In the framework of this scientific investigation, the utilization of single-cell multi-omics technologies has proven pivotal in effectively assaying and simultaneously evaluating distinctive molecular attributes arising from an individual cellular entity [1]. This innovative approach facilitates the measurement of multiple molecular species within the same cell, unlocking profound biological insights that surpass those attainable by analyzing separate molecular layers from distinct cells [2]. Moreover, the advancement of single-cell multi-omics technologies as a related discipline, along with comprehensive research in associated concepts, has led to the development of an expanding array of single-cell multi-omics technologies. Consequently, advancements in technologies are consistently improving the efficacy of these methodologies, while concurrently reducing their associated expenses.

One emblematic paradigm of these cutting-edge methodologies encompasses single-cell epigenomic sequencing, an innovative technique harnessed for the comprehensive examination of the epigenetic profile within individual cells. This methodology encompasses the analysis of various epigenetic phenomena, including DNA methylation patterns and histone modifications. Distinctive to single-cell epigenomic sequencing is its focus on the intricate epigenetic landscape within individual cells, circumventing the need for sequencing the entire genomic sequence and instead emphasizing the discernment of epigenetic modifications within the genome. By employing single-cell epigenomic sequencing, scientists can unlock invaluable perspectives regarding the inherent heterogeneity and diversity that exists at the epigenetic level among cellular populations.

Another illustrative exemplification resides within the realm of single-cell epigenomics sequencing, a vanguard concept that encompasses a more expansive scope of investigation. Within this paradigm, the epigenetic constitution within individual cells is comprehensively characterized, coupled with the sequencing of the genome in each individual cell. In addition to the meticulous scrutiny of epigenetic attributes, single-cell epigenomics sequencing adeptly captures the genome's sequence information within cells, thereby endowing a more holistic and comprehensive depiction of single-cell profiles. This interdisciplinary approach grants researchers the capacity to explore the intricate interplay between genomic and epigenetic landscapes, assessing their dynamic roles in cellular development, pathogenesis, and other fundamental biological processes.

In summary, a rapidly expanding range of single-cell multi-omics methodologies are gaining significant popularity in various scientific disciplines. Within this scholarly review, our objective is to offer a comprehensive outline of the latest breakthroughs in single-cell multi-omics techniques, elucidating their intricate connections with tumors and their growing applications in the field of cancer research. By emphasizing the crucial role played by single-cell multi-omics techniques, we aim to underscore their importance and relevance in advancing our comprehension and control of cancer-related phenomena.

## 2 The development of single-cell multi-omics techniques

### 2.1 The orientations

Due to the inherent heterogeneity in epigenetic profiles among cells, the trajectory of cellular differentiation can vary considerably, a phenomenon that becomes increasingly pronounced with the maturation and growth of the human body. As cells diversify and assume distinct functions, profound challenges are presented to the fields of human development, stem cell biology, and related disciplines. In light of cell heterogeneity, single-cell analysis has become an expanding area of research within life sciences and precision medicine. Recent advancements in analytical platforms and instrumentation have revitalized this field, paving the way for the emergence of highly sensitive, high-throughput, and multiplexed single-cell omics techniques. These omics tools serve as a bridge connecting molecular alterations at a cellular level with observable cell behavior, thus facilitating a deeper comprehension of disease progression processes [3]. With the aid of single-cell multi-omics techniques, it becomes feasible to tackle numerous intricate problems by scrutinizing gene expression patterns within individual cells, thereby unraveling the underlying behavior, mechanisms, and interplay between individual cells and the human body [4].

### 2.2 The breakthroughs

Single-cell multi-omics methodologies encompass a broad range of techniques and approaches that facilitate the comprehensive characterization of cell state and activity. By simultaneously integrating diverse single-modal omics methods, such as those capturing information on the transcriptome, genome, epigenome, proteome, metabolome, and other emerging omics, these techniques provide unparalleled insights. Collectively, these advancements are revolutionizing the landscape of molecular and cellular biology research. With the advancement of research and technology, a plethora of diverse single-cell multi-omics techniques have been conceived, developed, and refined, finding utility across various scientific domains [5].

When applied to stem cell or developmental biology research, these techniques exhibit the ability to delineate the intricate regulatory networks driving cellular differentiation [6]. When applied to studies in stem cell or developmental biology, these methodologies demonstrate the capacity to elucidate the intricate regulatory networks governing cellular differentiation. The specific examples below highlight the successful implementation of these techniques. Esteban et al. [7] harnessed multiple single-cell and multi-omics technologies to generate human pluripotent stem cells from somatic cells that faithfully recapitulate the developmental stage of 3-day-old fertilized eggs. Notably, these human cells represent the most nascent population to date cultivated in vitro, signifying a groundbreaking achievement in regenerative medicine. This work exemplifies the convergence of regenerative medicine and single-cell multi-omics technology. Furthermore, Tang et al. [8] employed a series of single-cell multi-omics techniques to longitudinally analyze seven test-tube babies born after chimeric blastocyst transfer. Through this investigation, the study offered the first comprehensive evaluation of test-tube babies derived from chimeric embryos at a single-cell resolution and from a multi-omics standpoint. The findings revealed a high rate of successful development into healthy babies and addressed the issue of chromosomal mosaicism during the embryonic stage. Consequently, this research advances our understanding of trophectoderm biopsy-based diagnosis of chimeric embryos, offering novel insights into this field.

In the field of immunology, the utilization of single-cell multi-omics methodologies enabled the elucidation of the epigenetic regulatory mechanisms underlying immune cell activation and differentiation [9]. A typical example of this is that Lan et al. [10] revolutionized single-cell transcriptome sequencing with FIPRESCI, a technique that integrates combinatorial marker technology and optimizes microfluidic platforms. By improving cell throughput over tenfold and reducing costs, FIPRESCI overcomes limitations of conventional methods, enabling multi-modal analysis of the single-cell transcriptome. Another example is that Xue et al. [11] explored liver cancer heterogeneity at the single-cell level using various sequencing techniques. They defined five immune microenvironment subtypes of liver cancer (TIMELASER) with single-cell precision for the first time. Their research delved into cellular composition, chemokine receptor-ligand network, cell spatial distribution, and specific genomic characteristics. Extensive validation confirmed the tumor-promoting mechanisms of CCL4+ and PD-L1+ TAN. The authors also constructed a mouse liver cancer model and demonstrated the potential of targeting tumor-associated neutrophils as a novel immunotherapy strategy.

Moreover, the significant advancement in single-cell multi-omics technology has revolutionized the analysis of complex structures within rare tumor cells, enabling a comprehensive understanding of tumor occurrence, progression, and therapeutic strategies. These cutting-edge techniques have proven to be indispensable in the identification of distinct tumor subtypes and the exploration of critical pathways involved in tumor cell development [12]. Notably, Nam et al. [13] conducted a groundbreaking study in which they employed single-cell multi-omics techniques and integrated multiple data models to investigate the evolution of cancer. Their research shed light on the intricate interplay between genetic and non-genetic factors in somatic cell evolution, ultimately revealing that cancer is a result of complex interactions. Furthermore, their work offered valuable experimental insights for the ongoing research and development of single-cell multi-omics technology.

**Fig. 1:**
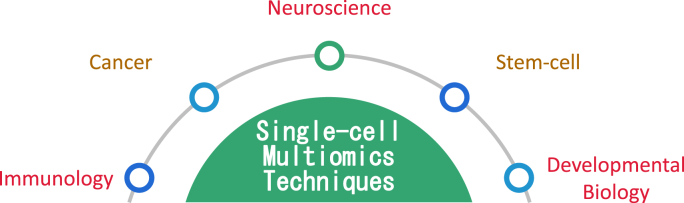
As single-cell multi-omics techniques develop, they're applied to various domains, such as immunology, cancer, neuroscience, developmental biology, etc. These applications will help to solve some problems in those experiments that are still in the bottleneck.

Last but not least, within the field of neuroscience, these methodologies are utilized to discern a multitude of intricate and variable subcategories of neuronal cells [14] while concurrently unraveling the molecular complexities governing diseases related to the nervous system [15] (Fig. 1). Mathys et al. [16] disclosed cellular and molecular connections related to cognitive function, dementia pathology, and resistance to AD pathology by means of a sequence of techniques entailing single-cell multi-omics. Furthermore, this investigation played a significant role in enhancing and advancing the methodologies employed in single-cell multi-omics. Additionally, in the exploration of the diversification and kinetics of precursor cells throughout the process of bodily aging, Lu et al. [17] in conjunction with neonatal cell markers and single-cell combinatorial indexing, have devised TrackerSci, a novel single-cell omics methodology to characterize the transcriptome and unbound chromatin of proliferating precursor cells in vivo—ultimately uncovering a multitude of diverse precursor cell classifications and their epigenetic attributes. The kinetics pertaining to the proliferation and differentiation specific to certain cell types associated with aging were quantified, and genes responsible for age-related regulation were identified.

### 2.3 The progressions and discoveries

Through single-cell multi-omics techniques, it is possible to determine the presence of heterogeneity among cells derived from the same or distinct tissues, organs, or systems. As cellular fate is intricately governed by the genetic material, the essential distinctions which commonly manifest in the genome, transcriptome, epigenome, and various other levels play a crucial role [18]. To enhance our understanding of organismal functional mechanisms and identify more efficacious disease treatment approaches, the utilization of this particular technique offers a distinct advantage. It circumvents the challenges associated with bulk sample homogenization, which may inadvertently conceal the heterogeneity within individual cells. Moreover, it enables comprehensive exploration of cellular states in both healthy physiological conditions and disease aberrations, all accomplished at the single-cell level concurrently.

Initially, the early single-cell multi-omics techniques encountered several limitations, including high costs and technical complexities, which hindered their widespread adoption. During the nascent stages, these techniques could only detect a limited repertoire of genes within a single cell. However, the advent of combining chip technology addressed these challenges effectively.

Due to the early single-cell, multi-omics techniques exist lots of limitations, high costs, and difficulty, it was not widely used at the beginning. In the very beginning, single-cell multi-omics techniques can only detect a small number of genes from a single cell, then the combining chip technology was born. In 2009, Professor Tang Fuchou devised a pioneering technique that merged single-cell transcriptome analysis with high-throughput sequencing, unveiling a pathway towards continuous improvement in gene detection throughput. This remarkable development eventually led to the formulation of the single-cell trace mRNA sequencing scheme [19]. However, these techniques were initially limited in their applicability to only a few cell types as samples. In 2015, McCarroll et al. [20] introduced a solution that revolutionized the field, centered around the utilization of microdroplet encapsulation of single cells and the capture of magnetic beads. Fast forward to 2023, Unlu Yazici, M. et al. [21] has recently introduced an innovative approach for multi-omics data analysis, leveraging machine learning (ML) techniques. This integrative methodology aims to group and score these groups using well-established biological knowledge. By exploring the intricate interactions between the three omics datasets, this integrated data environment holds promise for enhancing prediction, diagnosis, and treatment of cancer, a highly complex disease. Moreover, the proposed method bridges the gap in interpreting disease mechanisms that underlie disease onset and progression.

In addition to the aforementioned examples, recent years have witnessed the rapid development of numerous single-cell technologies. Large-scale initiatives including the Human Cell Atlas, the Mouse Cell Atlas, the Mouse RNA Atlas, the Mouse ATAC Atlas, and the Plant Cell Atlas have contributed significantly to the generation of extensive single-cell omics datasets [22].

**Fig. 2:**
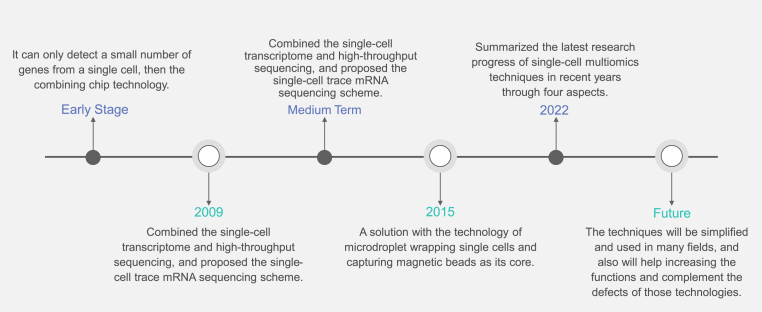
The timeline shows the key developments and the changing of single-cell multi-omics techniques.

Due to the rapid advancements in cell isolation technology and high-throughput sequencing, there has been a significant improvement in detection efficiency. Moreover, the capacity to detect a larger number of cells in a single experiment has substantially increased. Consequently, the resulting data now more accurately reflects the true cellular state within tissues, organs, and other biological systems. This enhanced capability is particularly advantageous for exploring and uncovering novel cell types [23]. Wen and Tang provided a comprehensive overview of the recent advances in single-cell multi-omics techniques, with a specific emphasis on their application in human stem cell research. In their analysis, they covered four key aspects to highlight the latest research progress in this field [24] (Fig. 2).

## 3 Applications in precision on-omics and cancer therapeutics

**Fig. 3:**
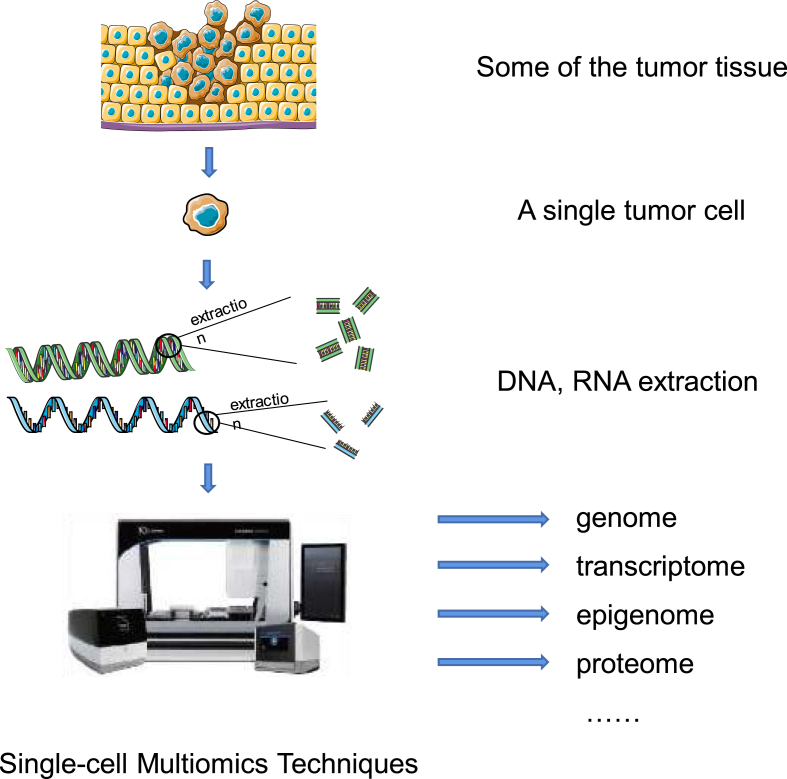
This is the principle of single-cell multi-omics techniques while being used in oncology. It can overcome the limitation of the traditional bulk genome and transcriptome analysis, and also have an elaborate dissection of the cellular and molecular level.

In recent years, the widespread availability of genomic data obtained from samples obtained from patients has brought about new opportunities in the analysis of gene mutations and alterations. Consequently, the visualization and subsequent identification of genes that have undergone mutation in large sets of patients have become crucial tasks that provide valuable insights into personalized intervention strategies [25]. The progress in next-generation sequencing and mass spectrometry has necessitated the integration of biological features to comprehend a system holistically. Biological aspects, including the transcriptome, methylome, proteome, histone post-translational modifications, and the microbiome, all exert an influence on the host response to various diseases and cancers [26]. By utilizing single-cell multi-omics techniques, we can examine each individual cell at the levels of the genome, transcriptome, epigenome, and proteome, overcoming the limitations of traditional bulk genome and transcriptome analyses and achieving a comprehensive understanding of cellular and molecular processes (Fig. 3).

### 3.1 Hematologic malignancy

#### 3.1.1 Multiple myeloma, MM

MM is a malignancy characterized by the proliferation of abnormal plasma cells within the bone marrow, which stands as the second most prevalent hematological cancer, displaying a diverse array of subtypes and therapeutic approaches (Table 1).

**Table 1:** The concept of single-cell epigenomics and single-cell multi-omics techniques have made abundant progress in many fields, especially in the field of cancer. Through the single-cell multi-omics techniques, the complex tumor microenvironment in the dimension of single-cell can be analyzed, which reveals the mechanism of the difference in patient prognosis and drug response. They also play an important role in revealing the tumor microenvironment, tumor heterogeneity, drug efficacy, and other studies.

Cancer type		Definition	Main application achievements of single-cell multiomics techniques
Hematologic Malignancy	Multiple Myeloma	A malignancy characterized by the proliferation of abnormal plasma cells within the bone marrow	1. Risk stratification scores and treatment response prediction. (Ovejero et al. Explor Target Antitumor Ther (2021))
			2. Reveal a novel molecular tolerance pathways.(Cohen et al. Nat Med (2021)
			3. Identify PPIA as an effective therapeutic target.(Cohen et al. Nat Med (2021))
			4. A basis to learn drug resistance through single-cell multi-omics.(Cohen et al. Nat Med (2021))
			5. The potential of exploring clonal evolution patterns and advance precision medicine. (Ankit et al. Nat Rev Clin Oncol (2022))
	Myeloproliferative Neoplasms	A group of blood disorders characterized by the abnormal and uncontrolled proliferation of bone marrow cells	1. Provide some important findings in the pathological mechanism and treatment mechanism of MPNs.(Egeren et al. Cell Stem Cell (2021))
			2. Determined the cell types and fate bias of hematopoietic lineage progenitors.(Egeren et al. Cell Stem Cell (2021))
			3. Revealed megakaryocyte-biased hematopoiesis in Myelofibrosis and identify mutant clone-specific targets. (Psaila et al. Mol Cell (2022))
Lymphoma	Hodgkin Lymphoma	A rare malignancy accounting for roughly 15 % of all lymphomas and mostly affecting young patients.(Momotow et al. J Clin Med (2021))	1. Verify the tumor cells the expression of cytokines and chemokines.(Steidl et al. Blood (2023))
			2. Reveal the complex interaction between the microenvironment and tumorigenesis. (Steidl et al. Blood (2023))
			3. Hold the promise to discovery novel immunotherapeutic approaches.(Steidl et al. Blood (2023))
			4. Identify distinct T cell subsets in Hodgkin lymphoma microenvironment. (Aoki T et al. Cancer Discov (2020))
	Diffuse large B-cell lymphoma	The most common subtype of non-Hodgkin lymphoma, which is an aggressive and fast-growing malignancy that primarily affects B lymphocytes	1. Discover DLBCL tumor cell heterogeneity at a single-cell level. (Ren et al. Blood (2018))
			2. Analyzed DLBCL's immune microenvironment. (Ren et al. Cell Death Dis (2021))
			3. Reveal the interactions with microenvironmental cells. (Ren et al. Cell Death Dis (2021))
			4. The influence of HBV on tumor cell survival and immune evasion.(Ren et al. Cell Death Dis (2021))
			5. Revealed the interaction network between DLBCL and microenvironment cells.(Ye, X. et al. Blood (2018))
	Mantle Cell Lymphoma	An infrequent subtype of lymphoma characterized by notable heterogeneity and a high likelihood of recurrence.(Armitage, J. O. et al. N Engl J Med (2022))	1.Revealed the complexity and interaction of the molecular mechanism of MCL.(Nadeu, F. et al. Blood (2020))
			2.Identify MCL's cellular markers.(Wang, L. et al. Cancer Biol Med (2020))
			3.Guide the individualized treatment and promote drug development.(Wang, L. et al. Cancer Biol Med (2020))
	Follicular Lymphoma	A malignancy of low-grade B-cells with the propensity to undergo transformation into a highly aggressive and life-threatening ailment.(Tamma R et al. J Clin Med (2022))	Constructed a single cell transcriptome map of human NHCs.(Abe, Y. et al. Nat Cell Biol (2022))
Neuroblastoma		A kind of out-cranial neoplasm which has the lowest five-year survival rate of any childhood cancer.	1.Discover that the tumor cells of neuroblastoma are originated from sympathetic neuroblasts that only exist in postnatal tumors of children.(Kildisiute, G. et al. Sci Adv (2021))
			2.Determine the cell types in the developing adrenal medulla and their lineage trajectories at different stages of embryonic and fetal development.(Jansky S et al. Nat Genet (2021))
			3.Provid a new perspective for in-depth understanding of the pathogenesis of neuroblastoma.(Dong R et al. Cancer Cell (2020))
Colorectal carcinoma		One of the most common malignant tumors the gastrointestinal tract.	1.Analyze the mechanisms underlying the molecular map of human colorectal cancer genesis and metastasis.(Tang F et al. Science (2018))
			2.Define a continuum of cell status and composition changes of malignant transformation of polyps into colorectal cancer.(Becker WR et al. Nat Genet (2022))
			3.Reveal the dynamic characteristics of immune cells in CRLM.(Wu, Y. et al. Cancer Discov (2022))
Ovarian Cancer		A malignant tumor of the ovary, which is one of the most common tumors in female reproductive organs.	1.Find mutations of previously uninvolved ovarian cancer.(Papp E et al. Cell Rep (2018))
			2.Characterize the human oviduct epithelial tissue, then find that tumor-typing is based on non-genetic heterogeneity, which was closely related to survival.(Hu Z et al. Cancer Cell (2020))
Breast Carcinoma		One of the "hidden killers" of women, whose incidence rate of female malignant tumors is the top.	1.Dissect the complex breast cancer microenvironment(Chung W et al. Nat Commun (2017))(Wagner J et al. Cell (2019)), which can reveal different cell subsets that influence the antitumor response.(Karaayvaz M et al. Nat Commun (2018))(Wang Q et al. Nat Commun (2019))
			2.Assess the genetic heterogeneity of tumor cells at single-cell resolution.(Vu TN et al. Bioinformatics (2019))
			3.Identify the phenotypic relevance of somatic genomic alterations as well as demonstrate their variable impact on the tumor ecosystem.(Ali HR et al. Nat Cancer (2020))
Cervical cancer, CC		CC that is caused by high-risk human papillomavirus (HPV) remains a significant public health problem worldwide.	1. The tumors with productive HPV integration are associated with higher E6/E7 proteins and enhanced tumor aggressiveness and immunoevasion.(Fan, J. et al. Cell Genom (2023))
			2. Indicated significant dysregulation of metabolic pathways in RIAIS patients.(Yanlan, C. et al. Aging (2022))
Bladder Cancer		The most common malignancy of the urinary system.(Flaig TW et al. J Natl Compr Canc Netw (2022))	1.Distinguished the IPRPs and revealed novel prediction model.(Xu, W. et al. Ann Med (2021))
			2.Clarify the prognostic significance of tumor mutation burden in bladder cancer and its correlation with important immune infiltrating cells.(Zhang C. et al. J Cell Physiol (2020))
			3.Describe the immune cell composition of bladder cancer microenvironment under different clinical conditions.(Wu S. et al. Small (2019))
Esophageal Cancer		A kind of gastroenteric tumor, which can be divided into esophageal squamous cell carcinoma and esophageal adenocarcinoma.	1.Propose potential targets for EC's treatment.(Wang Q et al. EBioMedicine (2021))
			2.Gat the single-cell ECSS large-scale mapping.(Zhang, X et al. Nat Commun (2021))
			3.Detail analysis of various types of cells in the tumor microenvironment and interactions between cells.(Zhang, X et al. Nat Commun (2021))
Liver Cancer		A kind of gastroenteric tumor, which can be divided into primary and secondary.	1. Accelerate the understanding of liver cancer biology and bring new therapeutic opportunities.(Fan J et al. Zhonghua Gan Zang Bing Za Zhi (2020))
			2. Reduced cellular acetyl-CoA and protein hypoacetylation promote liver cancer growth and dedifferentiation.(Wu Z et al. Mol Cell (2022))
			3. Provided integrated multi-omics landscape of LM.(Yang S et al. Gastroenterology (2023))
Lung Carcinoma		A kind of malignant neoplasms arising from the bronchial mucosa or glands of the lungs.	1.Revealed the mechanisms related to the enhancement of proliferation, metastasis and EGFR-TKI resistance in EGFR-mutant LUAD with ARID1A deficiency.(Sun et al. Cell Commun Signal (2023))
			2.CD74 played an important role in EGFR-TKI resistance.(Kashima Y et al. Cancer Res (2021))
			3.The numeric value of cancer-tissue’ stemness is well above the para-carcinoma tissue, and the tumor-cells’ stemness index is related to tumor stage.(Zhao M et al. J Cancer Res Clin Oncol (2020))(Zeng H et al. Front Genet (2020))
Prostate cancer		An epitheliogenic malignant tumor occurs in the prostate gland.	1.Identify the Dist-luminal-C cells as Luminal progenitor cells at the invagination tip.(Guo W et al. Nat Genet (2020))
			2.Reveal the change of tumor-cells at every development of prostate cancer microenvironment in different historical periods.(Chen S et al. Nat Cell Biol (2021))
			3.Find out that the molecular classification at single-celled levels of the prostate cancer as well as the tumor-cells subtypes associated with tumor progression markers.(Chen S et al. Nat Cell Biol (2021))
Renal Cell Carcinoma		A malignant tumor originating from the renal tubular epithelium and is classified according to pathological type.	1.Delineate RCC's transcriptome and epigenomic profiles.(Xie Q. et al. Cell (2022))
			2.Identify the regulatory factors that play important roles in the recognition between tumor cells and a variety of immune cells.(Xie Q. et al. Cell (2022))
			3.Identify different types of renal cells and renal tumor cells.(Young, M. D. et al. Science (2018))
			4.Reveal the precise cell classification and composition in renal tumors, as well as described the human renal cell map and malignant transformation of wilms tumor with nephrogenesis.(Young, M. D. et al. Science (2018))
			5.Find out that blocking WNT and NOTCH has the potential to treat RCC.(Fendler A et al. Nat Commun (2020))

Ovejero, S.et al. [27] suggested that integrating omics data and clinical histories enables risk stratification scores and treatment response prediction in patients upon diagnosis (Table 1). In addition to the aforementioned study, Cohen et al. [28] revealed novel molecular tolerance pathways in MM via single-cell multi-omics and clinical trials, identifying PPIA as a promising therapeutic target, offering a basis for future clinical trials to understand drug resistance in MM patients using single-cell multi-omics (Table 1). Moreover, Ankit et al. [29] emphasized the potential of genomics and single-cell multi-omics techniques to explore clonal evolution patterns and advance precision medicine for MM.

#### 3.1.2 Myeloproliferative neoplasms, MPNs

MPNs are a group of blood disorders characterized by the abnormal and uncontrolled proliferation of bone marrow cells [30].

Egeren et al. [31] applied cutting-edge single-cell multi-omics methods to study transcriptomes of isolated bone marrow cells. They identified nine cell types and observed lineage bias in hematopoietic progenitors. Using somatic mutations as reference, they reconstructed the cancer cell lineage tree, enabling precise tracking of cell progeny. This comprehensive framework greatly improved our understanding of cellular differentiation (Table 1). Shifting the focus to Myelofibrosis, Psaila et al. [32] similarly employed single-cell multi-omics techniques to delve into the investigation of this specific disorder. Their groundbreaking discoveries notably emphasized a significant bias towards megakaryocyte-derived hematopoiesis in the context of Myelofibrosis. Additionally, this comprehensive analysis led to the identification of specific targets specifically associated with the mutant clone, thereby opening new avenues for intervention and targeted therapies (Table 1).

### 3.2 Lymphoma

#### 3.2.1 Hodgkin lymphoma (HL)

HL is a rare malignancy accounting for roughly 15 % of all lymphomas and mostly affecting young patients [33](Table 1). The microenvironment in HL is distinguished by a notable abundance of non-malignant immune cells, whereas the presence of tumor cells is comparatively limited. These tumor cells originate from dysregulated clonal proliferation and malignant transformation of lymphocytes, undergoing extensive interactions with immune cells throughout their development [34]. Steidl et al. [35] utilized advanced single-cell multi-omics to analyze the cellular composition and spatial distribution in HL microenvironment. Their findings confirmed cytokine and chemokine expression in tumor cells, revealing the complex interaction between the microenvironment and tumorigenesis, holding the promise to accelerate biomarker discovery for novel immunotherapeutic approaches and to serving as future assay platforms for biomarker-informed treatment selection, including immunotherapies (Table 1). Furthermore, Aoki et al. [36] utilized single-cell multi-omics to identify distinct T cell subsets in Hodgkin lymphoma microenvironment, leading to a significant breakthrough. Their findings enhance our understanding of the disease and drive technological advancements and disease-centric research, propelling progress in the field (Table 1).

#### 3.2.2 Diffuse large B-cell lymphoma (DLBCL)

DLBCL is the most common subtype of non-Hodgkin lymphoma, which is an aggressive and fast-growing malignancy that primarily affects B lymphocytes [37].

Ren et al. discovered DLBCL tumor cell heterogeneity at a single-cell level using multi-omics in HBV-infected individuals in 2018 [38]. In 2021 [39], they analyzed DLBCL's immune microenvironment, revealing interactions with microenvironmental cells and the influence of HBV on tumor cell survival and immune evasion (Table 1). In another study, Ye et al. [40] found phenotypic heterogeneity in DLBCL using single-cell multi-omics, highlighting the impact of HBV infection on survival and immune evasion, offering insights for immunotherapy (Table 1). These groundbreaking studies have not only broadened our comprehension of the fundamental mechanisms in B-cell lymphoma but also facilitated the advancement and utilization of cutting-edge single-cell omics techniques.

#### 3.2.3 Mantle cell lymphoma, MCL

MCL is an infrequent subtype of lymphoma characterized by notable heterogeneity and a high likelihood of recurrence [41] (Table 1).

Scientific research by Nadeu et al. [42] uncovered molecular mechanisms in MCL using genome and epigenome analysis. The study discovered varied subtypes and patient outcomes, providing valuable insights into this complex disease (Table 1). Additionally, Wang et al. [43] used state-of-the-art single-cell multi-omics techniques to investigate immune evasion and drug resistance in MCL. This work identified new markers and revealed the involvement of these genes. The prognostic value was confirmed using public datasets. These findings offer insights for personalized treatment and drug development in MCL (Table 1). The aforementioned studies not only establish a theoretical framework for personalized therapy of MCL, but also offer substantial data support and limitless prospects for future advancements in single-cell multi-omics technology.

#### 3.2.4 Follicular lymphoma, FL

FL represents a malignancy of low-grade B-cells with the propensity to undergo transformation into a highly aggressive and life-threatening ailment [44] (Table 1).

For example, Abe et al. [45] created a detailed transcriptome map of non-hematopoietic cells in humans using single-cell multi-omics techniques. They found unique gene expression patterns in various subsets of these cells in follicular lymphoma (FL), shedding light on their interaction with cancer cells. This research improves classification and disease analysis in FL and suggests potential biomarkers for diagnosis and treatment. Overall, it deepens our understanding of lymphoma biology (Table 1).

### 3.3 Neuroblastoma

Neuroblastoma, a prevalent childhood malignancy predominantly impacting children below the age of five, exhibits the most unfavorable five-year survivability among all pediatric cancers. In an endeavor to unravel the molecular characteristics of neuroblastomas, Kildisiute et al. [46] employed single-cell multi-omics techniques to comprehensively investigate a substantial cohort of these tumors. Notably, their investigation revealed the exclusive presence of sympathetic neuroblasts within postnatal tumors of children, implying their potential as novel therapeutic targets (Table 1). To illuminate the lineage relationships and developmental dynamics of human adrenal medulla cells, Frank et al. [47] leveraged single-cell multi-omics approaches. Through their meticulous analysis, they successfully discerned distinct cellular populations within the developing adrenal medulla and meticulously delineated their lineage trajectories across diverse stages of embryonic and fetal development (Table 1). Furthermore, incorporating the power of single-cell multi-omics techniques, Wang et al. [48] conducted an in-depth exploration of the single-cell landscape of neuroblastomas in children. This pioneering endeavor yielded novel insights into the intricate pathogenesis underlying this devastating malignancy (Table 1).

### 3.4 Colorectal carcinoma, CRC

As Tang et al. [49] pioneered the usage of single-cell multi-omics methodologies in 2018 to elucidate the molecular events underlying the initiation and spreading of colorectal cancer (CRC), this technological approach has gained paramount significance in CRC investigations (Table 1). In a subsequent study, Michael et al. [50] utilized single-cell multi-omics techniques to investigate the transcriptomic and epigenomic profiles of individual cells derived from tissue samples, thereby shedding light on the continuum of cellular states and compositions that accompany the transformation of polyps into colorectal cancer (Table 1). Further expanding the repertoire of single-cell multi-omics tools, Wu et al. [51] extensively applied various strategies including single-cell transcriptome sequencing, spatial transcriptome sequencing, and multiplex immunofluorescence technology to comprehensively explore the dynamic attributes of immune cells within colorectal cancer liver metastases (CRLM) (Table 1).

### 3.5 Ovarian cancer

Ovarian cancer is a prevalent neoplasm that commonly affects the female reproductive system. In order to elucidate intricacies of this disease, Eniko et al. [52] utilized single-cell multi-omics methodologies to investigate various subtypes of ovarian cancer. This study unraveled previously unexplored mutations associated with ovarian cancer, thereby furnishing an extensive and broadly accessible trove of molecular information necessary for the advancement of therapeutic avenues in ovarian cancer management (Table 1). In a bid to decipher the intricacies of human oviduct epithelial tissue, Yau et al. [53] harnessed the potential of single-cell multi-omics techniques. Their findings unveiled that tumor classification is predominantly reliant on non-genetic heterogeneity, which exhibited a close correlation with survival rates. Consequently, they proposed a molecular classification approach that demonstrates remarkable stability in predicting prognosis (Table 1).

### 3.6 Breast carcinoma

Since single-cell multi-omics techniques can dissect the complex breast cancer microenvironment [54], which can reveal different cell subsets that influence the antitumor response [55] (Table 1). They can also assess the genetic heterogeneity of tumor cells at a single-cell resolution (Table 1). To sum up, single-cell multi-omics techniques can help elucidate the biological complexity of tumors. Furthermore, single-cell multi-omics techniques can be used to detect the emergence of drug-resistant cell subsets after treatment, which provided a great important clinical significance to the second-line treatment decisions of existing or new targeted drugs in breast cancer. By using single-cell multi-omics techniques to analyze and integrate the sampled data, Carlos Caldas and Bernd Bodenmiller [56] identify the phenotypic relevance of somatic genomic alterations as well as demonstrate their variable impact on the tumor ecosystem, which can be used as a marker for patient stratification and target for the development of new therapies (Table 1).

### 3.7 Cervical cancer, CC

CC caused by high-risk human papillomavirus (HPV) remains a substantial global public health challenge. Integration of HPV into the host genome can occur with or without transcription, resulting in the formation of viral-host fusion transcripts. Exploiting extensive large-scale, multi-omics datasets, Fan et al. [57] present compelling evidence that tumors with active HPV integration display heightened expression of E6/E7 viral oncoproteins, culminating in augmented tumor aggressiveness and evasion of the immune system (Table 1). In a similar scientific endeavor, Yanlan et al. [58] adopt an all-encompassing integrated multi-omics strategy, combining 1H NMR metabolomics and bioinformatics analyses, to probe into the pathogenic mechanisms driving recurrent intra-abdominal inflammatory syndrome (RIAISs). By scrutinizing the investigations, the authors propose a hypothesis delineating the disrupted metabolic pathways underpinning RIAISs, which is subsequently experimentally validated using western-blot analysis. The obtained results shed light on the profound perturbations occurring in metabolic pathways among individuals afflicted with RIAISs (Table 1).

### 3.8 Bladder cancer, BC

Bladder cancer stands as the preeminent malignancy within the urinary system [59] (Table 1). In the realm of bladder cancer research, Xu et al. [60] undertook a comprehensive, large-scale multi-omics analysis. Their study successfully discriminated Individualized Patient Response Profiles (IPRPs) and introduced a groundbreaking predictive model that surpasses conventional prognostic indicators, which demonstrates superior performance in accurately predicting the progression of bladder cancer and formulating more effective clinical strategies (Table 1). In a similar vein, Zhang et al. [61] conducted an examination focusing on a substantial quantity of bladder cancer samples using single-cell multi-omics techniques. Their investigation significantly clarified the prognostic significance of tumor mutation burden in bladder cancer, alongside its correlation with pivotal immune infiltrating cells. These findings serve as a valuable reference for the selection of subpopulations pertaining to individualized immunotherapy (Table 1). Furthermore, Wu's team [62] also employed single-cell multi-omics techniques to obtain single-cell transcriptome profiles of both cystitis glandulous and bladder cancer under diverse clinical conditions. With this approach, the team delineated the immune cell composition within the bladder cancer microenvironment, shedding light on biomarkers intimately associated with the biological processes and clinical prognosis of bladder cancer (Table 1).

### 3.9 Esophageal cancer, EC

EC can be categorized into two principal subtypes, namely esophageal squamous cell carcinoma (ESCC) and esophageal adenocarcinoma (EAC). In a recent study conducted by Wang's team [63], ESCC heterogeneity and the tumor microenvironment were thoroughly analyzed using single-cell multi-omics techniques. This investigation not only provided new insights into the complex nature of ESCC but also identified potential therapeutic targets for its treatment (Table 1). Similarly, Zhang, X et al. [64] utilized single-cell multi-omics techniques to analyze a representative sample group, resulting in a comprehensive mapping of ESCC at the single-cell level. The researchers performed detailed analyses of various cell types present in the tumor microenvironment and elucidated the interactions between these cells. Consequently, they provided a comprehensive description of the ESCC microenvironment and its immunosuppressive state. Furthermore, the researchers identified genes associated with patient survival in relation to the highly epithelial mucosa program (Table 1).

### 3.10 Liver cancer

Technological breakthroughs and revolutions, such as single-cell sequencing, genome editing, and innovative drug research and development, will greatly accelerate the understanding of liver cancer biology and bring forth new therapeutic opportunities [65]. Employing multi-omics methodologies, Park et al. [66] demonstrate that diminished cellular acetyl-CoA and protein hypoacetylation facilitate the proliferation of liver cancer cells and their transition into an undifferentiated state (Table 1). Furthermore, Yang, S.et al. [67] utilize various single-cell multi-omics techniques, including single-cell transcriptomic sequencing and multiplex fluorescent immunohistochemistry, to comprehensively map the multi-omics landscape of liver metastasis (LM), thereby unraveling prospective mechanisms underlying aggressive inter-metastatic subtypes and validating the regulatory functions of SLC2A1 in remodeling the tumor microenvironment in primary tumors as well as LM lesions (Table 1).

### 3.11 Lung carcinoma

Utilizing state-of-the-art single-cell multi-omics techniques, Sun et al. [68] unraveled the intricate molecular mechanisms modulating augmented proliferation, metastasis, and resistance to EGFR-TKI in EGFR-mutant LUAD cases with ARID1A deficiency (Table 1). In a similar vein, Kashima et al. [69] utilized employed state-of-the-art techniques to detect the single-cell transcriptome and chromatin accessibility (ATAC) profile in clinical tumor samples and drug-resistant EGFR-TKI cell models. Moreover, the concept of stemness in tumors emerges as a crucial determinant of tumor occurrence, progression, treatment response, and overall clinical outcome (Table 1). Both Zhao et al. [70] and Zeng et al. [71] elved into the tumor cells’ stemness index and its associated genes using cutting-edge single-cell multi-omics technologies, thereby offering valuable insights into patient prognosis. The comprehensive findings from these investigations collectively demonstrate significantly elevated cancer tissue stemness values compared to para-carcinoma tissue, further underscoring the correlation between tumor stage and the tumor cells' stemness index (Table 1).

### 3.12 Prostate cancer, PC

By utilizing single-cell multi-omics methodologies, Gao [72] discovered that the Dist-luminal-C cells exhibited self-renewal capacity. Additionally, they classified these cells as Luminal progenitor cells located at the invagination tip, thus shedding light on the lineage hierarchy within the prostate gland (Table 1). By employing single-cell multi-omics approaches, Ren's team [73] elucidated the intricate dynamics of tumor cells during various historical stages of prostate cancer progression. Moreover, they delineated the molecular classification of prostate cancer at the single-cell level and identified distinct tumor-cell subtypes associated with markers of disease progression. These findings hold profound implications for the advancement of novel treatment strategies in prostate cancer and the identification of tumor biomarkers (Table 1).

### 3.13 Renal cell carcinoma, RCC

RCC is a malignant neoplasm originating from the renal tubular epithelium and is categorized based on pathological subtype.

Xie et al. [74] employed single-cell multi-omics techniques to analyze data and elucidate the transcriptome and epigenomic profiles of RCC. Moreover, they delineated the regulatory factors crucial for the crosstalk between tumor cells and diverse immune cell populations, thereby unraveling the heterogeneity of RCC, as well as unveiling promising therapeutic targets (Table 1). By leveraging single-cell multi-omics methods, Young et al. [75] have comprehensively examined and identified distinct renal cell types, including renal tumor cells. They revealed a precise characterization of cell composition within renal tumors, described the human renal cell map, and explored the malignant transformation of Wilms tumor during nephrogenesis (Table 1). Fendler et al. [76] employed single-cell multi-omics techniques to identify RCC stem cells and subsequently discovered the potential of WNT and NOTCH inhibitors as therapeutic agents for RCC. This finding represents a significant contribution to personalized treatment strategies for clear RCC (Table 1).

## 4 Live cell imaging and protein detection in single-cell multi-omics

In recent years, the discipline of single-cell multi-omics has primarily concentrated on elucidating cellular heterogeneity through RNA expression analysis. However, in order to attain a more comprehensive comprehension of cellular behavior and functionality, it is imperative to investigate additional dimensions of the cellular landscape, encompassing protein expression and dynamic live-cell imaging. This chapter will delve into the integration of live-cell imaging and protein detection methodologies within the realm of single-cell multi-omics investigations.

### 4.1 Live cell imaging in single-cell multi-omics

#### 4.1.1 Principles of living cell imaging

Live-cell imaging [77] encompasses a variety of methodologies aimed at capturing images of cells in their vibrant and active state, presenting two main options: a single static image or a time-lapse series of images. By providing real-time monitoring of dynamic cellular processes, this technology allows researchers to gain valuable insights. It enables the examination of cell behavior, including but not limited to cell division, migration, and response to external stimuli. The integration of live-cell imaging into research practices holds the potential to enhance the development of biologically significant assays, thus improving the prediction of human responses to emerging drug candidates.

Currently, the techniques employed for live-cell imaging span a range of approaches, including time-lapse microscopy, fluorescence recovery after photobleaching (FRAP), high-content screening, fluorescence resonance energy transfer (FRET), and fluorescence in situ hybridization. These techniques, among others, contribute to the diverse toolbox available for live-cell imaging research. The data and experimental steps presented in this paper are sourced from Molecular Devices (https://www.moleculardevices.com.cn/applications/live-cell-imaging).

##### 4.1.1.1 Prerequisites for live-cell imaging

To successfully conduct live-cell imaging, certain conditions must be satisfied. These prerequisites ensure optimal cell viability and imaging quality, facilitating accurate observation and analysis of dynamic cellular processes. The key requirements include.

(1) The physiological conditions of the samples were maintained

In order to ensure optimum sample vitality and health during sample preparation and imaging, it is imperative to maintain the samples under conditions that closely resemble their physiological environment. This can be achieved by employing appropriate cell media, including buffer and growth serum, along with suitable culture vessels and aseptic techniques. A constant temperature of 37 °C, with a pH of 7.4, was maintained using a bicarbonate (HCO3-) and dissolved carbon dioxide (CO2) buffer system. The choice of culture vessel is also a critical factor, with plastic vessels generally promoting better cell growth, while glass vessels offer superior high-power optical quality. Managing the risk of bacterial contamination is of utmost importance, making the use of antibiotics a common approach. However, if a specific protocol necessitates the avoidance of antibiotics, extra care must be taken to ensure the proper preparation and maintenance of the culture process.

(2) Protect cells from phototoxicity

The utilization of fluorescence imaging in cellular studies necessitates careful consideration of potential implications on cellular homeostasis caused by phototoxicity, which may arise from various sources. Intracellular organic molecules, such as porphyrins and flavins, possess light-absorbing properties that, in the presence of oxygen, can degrade and give rise to reactive oxygen species, leading to cellular damage. Additionally, fluorescent dyes themselves can generate free radicals when interacting with oxygen while in an excited state. To mitigate phototoxicity, the following straightforward yet effective measures can be implemented.

a. Employing fluorophores with excitation spectra characterized by lower energy (longer wavelength).
b. Minimizing frame rates, particularly during extended experimental durations.
(3) Appropriate frame rate

When observing changing activity, we need to ensure that the image frame rate is appropriate for tracking these events. Too low a frame rate means a loss of temporal resolution and a poor representation of the process of interest in the record file. Conversely, too high a frame rate increases the risk of phototoxicity, resulting in the production of larger files. But if the activity to be recorded is so fast that the required frame rate causes phototoxicity, in this case it is very helpful to improve the sensitivity of the camera to achieve satisfactory results using the required frame rate at lower excitation light intensities and lower gains.

##### 4.1.1.2 Basic steps for living-cell imaging

(1) Cell Seeding: Cells were seeded into the designated laboratory vessels, such as chamber slides, Petri dishes, or microplates.
(2) Cell Treatment: Cells were subjected to the desired intervention, including the application of specific compounds, RNA interference (RNAi), etc.
(3) Fluorescent Labeling: If necessary, cells were appropriately labeled with the required fluorophore, such as a fluorescent dye or fluorescent protein.
(4) Environmental Regulation: For extended acquisition periods or live cell time-lapse experiments (continuous or non-continuous), comprehensive environmental control, encompassing air, temperature, and humidity, was meticulously maintained.
(5) Image Acquisition: The experimental plate was positioned within an automated imaging system, initiating the collection of live cell images.
(6) Image Analysis: Quantitative analysis of the acquired live cell images was performed employing dedicated Cell Imaging analysis software.

#### 4.1.2 Applications in cancer

Live cell imaging can be employed to investigate the heterogeneity of tumor cells within the microenvironment. In this study, we aim to elucidate how continuous observation of cellular dynamics can enhance our comprehension of tumor progression, metastasis, and therapeutic strategies.

##### 4.1.2.1 Elucidate the divergent spatial arrangement of extrachromosomal DNA elements and transcriptionally active extrachromosomal DNA clusters within tumor cells

In early January 2022, Eunhee Yi et al. [78] employed a CRISPR-based methodology to selectively label extrachromosomal DNA (ecDNA) in viable cells using distinctive ecDNA breakpoint sequences. The investigation revealed that the ecDNA copy number within individual cells adhered to a Gaussian distribution pattern, both in vitro tumor cell lines and glioblastoma specimens from patients. These findings indicate non-uniform segregation of ecDNA during mitosis. Subsequent to mitotic division, ecDNA exhibited a propensity to aggregate, coexisting with RNA polymerase II to facilitate the transcription of oncogenes. This study furnishes direct empirical evidence for the heterogeneous segregation of ecDNA through direct observation, while shedding light on a novel mechanism by which ecDNA participates in tumorigenesis.

##### 4.1.2.2 Elucidate the impact of pharmacological agents on the induction of autophagy in drug-resistant cancer cell lines

Despite the increasing success rates in cancer treatment, there remains a portion of patients who experience recurrence due to the loss of control over cancer cells by the original drug therapy and the emergence of drug resistance. Live cell imaging presents a unique opportunity to investigate the demise of reactivated "drug-resistant" cancer cells. In 2019, Liu Liang et al. [79] utilized advanced microscopy techniques, including Delta Vision wide-field reduced deconvolution, to elucidate the impact of lotus extract-induced compound Neferine on autophagy in drug-resistant cancer cells. These findings open up prospects for the future application of Neferine as a therapeutic agent targeting drug-resistant cancer cells.

##### 4.1.2.3 Reveal the structure of active rDNA chromatin in the nucleolus

In cases of malignant cancer cell proliferation characterized by heightened protein demands, ribosomes undergo significant amplification and assume a pivotal role. Recent years have witnessed the emergence of ribosome biogenesis inhibition as a novel strategy in cancer therapy. Leonhardt et al. [80] utilized Delta Vision OMX 3D-SIM microscopy to elucidate the intricate architecture of actively transcribed rDNA chromatin within the nucleolus, thereby pinpointing potential targets for precise cancer treatment. The continual advancements in ultra-high-resolution imaging techniques have considerably enhanced our comprehension of cellular processes at the individual-cell level. These techniques offer an enticing alternative to electron microscopy, compensating for the disruption in nucleolar organization and unraveling the salient characteristics of the active rDNA chromatin unit and its three-dimensional organization inside the nucleolus, while simultaneously preserving tissue viability.

##### 4.1.2.4 Promote the development of drugs against highly aggressive tumors

High throughput screening (HTS) technology is widely recognized for its efficiency in cancer drug development. However, a lack of HTS models for highly migratory cancer cells has been a challenge. Kim Young-Tae et al. [81] addressed this gap by introducing a groundbreaking approach using IN Cell Analyzer high-content imaging analysis. Their method enabled tailored high-throughput screening for migratory cancer cells, investigating the impact of drug molecules on migration speed. By accounting for physiological constraints, including tissue architecture and vasculature, they revealed the intricate interplay between drugs and cancer cells. This study highlights the potential of HTS methods in expediting therapeutics for aggressive tumors.

##### 4.1.2.5 Reveal the structure of tumor microenvironment (TME)

TME comprises a complex immune network crucial for tumor development. In a groundbreaking study, Chakra et al. [82] utilized SpAn and Cell DIVE multi-label imaging to analyze 55 biomarkers in rectal cancer tissue. This novel approach enables simultaneous biomarker marking, spatial information acquisition, and prediction models for cancer recurrence. These advancements offer new opportunities for precision medicine in the characterization and treatment of colorectal cancer (CRC).

### 4.2 Protein detection in single-cell multi-omics

#### 4.2.1 Cytometry by time-of-flight (CyToF)

CyToF, represents an innovative technology enabling high-dimensional analysis of protein expression at the single-cell level [83]. In this section, we will provide a comprehensive overview of CyToF, encompassing its underlying principles, notable advantages, and extensive applications in the field of oncology. Emphasizing its impact on tumor research, we will elucidate the pivotal role CyToF plays in unraveling the intricacies of neoplastic pathologies.

##### 4.2.1.1 The principle [84]

CyToF, a cutting-edge technology, merges the experimental platforms of flow cytometry and mass spectrometry, capitalizing on the strengths of both techniques. By effectively combining the single-cell analysis capabilities of conventional flow cytometry with the high-resolution advantages of time-of-flight mass spectrometry, CyToF revolutionizes the field. Leveraging metal-labeled antibodies as probes for antigen detection, CyToF enables high-throughput analysis at the single-cell level, facilitating simultaneous assessment of various parameters while elucidating distinct protein targets. By seamlessly integrating the traditional flow cytometry principles with mass spectrometry, CyToF inherits the velocity and efficiency of flow analysis while surmounting the inherent limitations of spectral overlap encountered in fluorescence-based flow cytometry approaches.

##### 4.2.1.2 Advantages [85]

(1) Abundance of detection channels: The ICP-TOF mass spectrometry device, equipped with a mass spectrometry flow meter, offers a wide range of atomic weight detection (typically 75–210Da). Consequently, it enables simultaneous measurement of numerous parameters, reaching into the hundreds.
(2) Non-interfering channels, eliminating the need for compensation calculations: The ICP mass spectrometry device possesses exceptional resolution capabilities, thereby allowing for clear differentiation of metal elements used as labeling agents. This crucially resolves the long-standing issue of "fluorescence string color" encountered in traditional flow techniques.
(3) Ample metal tags with minimal background effects: Metal tags are covalently linked to antibodies via polychelates, exhibiting an extremely low nonspecific binding rate with cellular components. Furthermore, the presence of lanthanide metals, which function as the metal tags within cells, is nearly absent, leading to an exceptionally low signal background.
(4) Enhanced sensitivity and stability: The mass spectrometry flow technology leverages the power of high-speed analysis and high resolution. As a result, it delivers highly sensitive results, ensuring data reliability.

##### 4.2.1.3 Application in tumors

###### 4.2.1.3.1 In-depth analysis of the tumor immune system map

Merad et al. [86] developed a barcoding technique that affixes distinct metal isotopes to cells in individual samples, enabling the pooling of samples for simultaneous analysis of cells from three different tissue types. This barcoding strategy was integrated with multiscale immune profiling to construct a comprehensive immune cell map, facilitating the identification of tumor-induced alterations.

###### 4.2.1.3.2 Patient stratification and response prediction for cancer immunotherapy

Krieg et al. [87] utilized high-dimensional single-cell mass spectrometry to analyze immune cell subsets in melanoma patients' peripheral blood. Using three antibody panels, they generated a wealth of valuable data from limited samples, supporting biomarker exploration. Notably, they found that monocyte abundance accurately predicted PD-1 inhibitor treatment response.

###### 4.2.1.3.3 The immune profile of fibroblasts with anti-tumor immunity was drawn

Cancer-associated fibroblasts (CAFs) play a crucial role in tumor progression via matrix remodeling, immune evasion, and drug resistance. Knowledge of fibroblast lineages and their association with diseases remains limited. Using CyTOF, Jorgensen et al. [88] analyzed stromal composition in tissues and tumors. They discovered heterogeneous mesenchymal phenotypes and coordinated interactions between mesenchymal cells and immune subsets in pancreatic adenocarcinoma. This research unveils distinct fibroblast lineages and highlights the importance of mesenchymal-immune cell interactions in inhibiting tumor growth. This research lays the groundwork for further investigations aimed at elucidating the intricate interplay between fibroblast lineages, immune cells, and tumor microenvironment dynamics, potentially providing novel therapeutic targets and strategies for cancer treatment.

#### 4.2.2 Other protein detection methods

In addition to CyTOF, we will briefly discuss alternative protein detection methodologies that can be incorporated into single-cell multi-omics investigations. These include liquid chromatography-tandem mass spectrometry (LC-MS/MS), protein proximity ligation assay (PLA), and Single-cell immunoblotting technique(scWestern). These techniques augment the repertoire utilized to delineate the proteomic landscape of individual cells.

##### 4.2.2.1 LC-MS/MS [89]

LC-MS/MS offers a powerful analytical approach by combining the superior separation capability of chromatography with the exceptional selectivity, specificity, and sensitivity of mass spectrometry using mass-to-charge ratio detection. Furthermore, LC-MS/MS effectively circumvents the issue of nonspecific interference typically encountered in immunoassays. This methodology proves especially advantageous for the analysis of low molecular weight compounds, including but not limited to hormones, amino acids, vitamins, and other similar substances.

#### 4.2.3 2PLA [90]

PLA, enables the localization and assessment of protein-protein interactions with high spatial resolution (within distances less than 40 nm) at endogenous protein concentrations. This innovative technique capitalizes on the utilization of specific antibodies, which can recognize and bind to the two proteins of interest either directly or indirectly. To further enhance the assay, DNA primers that are covalently linked to these antibodies are employed. Subsequent hybridization and PCR amplification steps, coupled with the utilization of fluorescent probes, allow for the visualization of spatially distinct fluorescence signals under a fluorescence microscope, thus facilitating the detection of protein proximity.

##### 4.2.3.1 scWestern

In a recent scientific study, Ding et al. [91] introduced a novel technology known as scWestern, which relies on a tetrazolium modified photosensitive hydrogel. This technology demonstrates high sensitivity and rapid response capabilities, making it suitable for single-cell immunoblotting. The experimental workflow involved chemical lysis of cells, followed by separation of the released proteins using SDS-PAGE electrophoresis. Subsequently, a short duration of ultraviolet excitation (∼60s) was used to activate the photosensitive gel, enabling cross-linking of proteins within the gel matrix. By harnessing the molecular sieve effect of SDS-PAGE, scWestern ensures protein detection specificity based on molecular weight and antigen-antibody recognition. It enables the identification of cell surface proteins, transmembrane proteins, intracellular and nuclear proteins, as well as discrimination of epigenetically modified proteins. Moreover, scWestern enables high-throughput and multi-index co-detection, facilitating the study of complex cell populations at the single-cell resolution. This innovative technique holds significant promise for various applications, including drug screening and investigations into cell heterogeneity.

### 4.3 Live-cell imaging and protein detection are integrated with multiple omics data

The integration of multi-omics data aims to identify cell types accurately [92]. This comprehensive approach enables a better understanding of differentiation paths, gene regulation, intercellular interactions, spatial organization, cell lineage, and clonal dynamics. By analyzing multiple molecular layers, we can uncover the underlying causes of cell phenotypes [93]. However, to effectively integrate high-dimensional data, it is crucial to merge live-cell imaging [77] and protein data with genomic, transcriptomic, and epigenomic data. This section will discuss strategies for consolidating data to grasp cellular heterogeneity [94].

#### 4.3.1 Single cell transcriptomics combined with mass spectroscopy-based proteomics

Protein markers with potential were initially identified through proteomics analysis. Subsequently, the cell types associated with these candidate proteins were investigated at the single-cell level by determining their corresponding gene expression profiles. This research concept is widely employed in the field. As an example, in 2019, Angelidis et al. [95] utilized single-cell transcriptome and protein profiling to measure alterations in cellular activity across 30 distinct cell types. Through integrative analysis, they generated a reference map of aging-related cells in the lung by predicting the cellular origins of regulatory proteins involved in the aging process.

#### 4.3.2 Paired-tag

In order to generate comprehensive maps of chromatin states and transcriptomes at the cell type level within complex tissues, an integrated analysis of histone modifications and transcriptomes in single cells was performed. Zhu et al. [96] developed a high-throughput assay that enables simultaneous detection of RNA expression and DNA tag sequencing in individual cells, thereby facilitating the integration of histone modifications and transcriptomic information at the single-cell level. This innovative approach offers a valuable tool for investigating chromatin states and single-cell transcriptomes in the context of complex tissues.

#### 4.3.3 MaxFuse

In the study conducted by Chen et al. [97], they present an innovative approach called MaxFuse, which involves the matching of X-modality via fuzzy smoothed embedding for cross-modal data integration. Through the utilization of iterative coembedding, data smoothing, and cell matching techniques, this method effectively leverages all available information within each modality to achieve a seamless and high-quality integration, even in cases where features exhibit weak associations.

### 4.4 Future developments and potential applications

Single-cell multi-omics has emerged as a critical approach for comprehending the intricacies of biological processes, particularly in rare diseases and cell types [98]. This technology integrates diverse omics features within a single cell, facilitating the accurate unraveling of complex molecular regulatory networks and their intra-cellular interactions [99]. The synergy of advanced bioinformatics algorithms enables the exploration of intercellular heterogeneity in complex tissues and the revelation of crucial mechanisms modulating cell function. Future advancements in single-cell multi-omics technology will emphasize the refinement of existing methodologies and the development of novel techniques [100]. Optimization of conventional practices encompasses database construction methodologies, sequencing technologies, and algorithmic tools. Furthermore, it encompasses experimental design optimization, automation of experimental procedures, and enhancement of experimental efficiency. Researchers can explore solutions from multidisciplinary fields such as biology, chemistry, and physics to better detect macromolecular characteristics. Additionally, novel computational methods and analytical tools transcend experimental limitations, facilitating the discovery of unexplored biological processes [101]. For instance, quasi-time series analysis aids in understanding cell differentiation trajectories during early embryonic development [102]. The novel formula pays attention to imperceptible aspects of gene expression regulation, including the three-dimensional structure of DNA [103], non-coding RNA [104], and intracellular proteins [105]. Nevertheless, the current momentum of development in single-cell multi-omics sequencing technology drives researchers to continuously enrich the single-cell sequencing toolbox and witness increasing competition among various omics methods. In the future, researchers must select stable, universal, cost-effective, and commercialized methods from an array of similar alternatives to expand their practical applications further.

## 5 The application of single-cell multi-omics in other fields

Single-cell multi-omics analysis reveals the immune response by capturing genomic, transcriptomic, and proteomic data, offering insights into immune cell subtypes, phenotypes, and activities [[106], [107], [108]]. This informs the understanding of tumor immunity, immune cell impact on therapies, and enables identification of distinct tumor subclones and intercellular interactions. Overall, these studies underscore the invaluable contributions of single-cell multi-omics technology in unraveling the complexities of immunological processes and shedding light on disease mechanisms.

### 5.1 The application of single-cell multi-omics in tumor heterogeneity

A neoplasm is an intricate blend of malign cells, immune cells, and stromal cells exhibiting tumoral heterogeneity. The neoplastic microenvironment encompasses both pro-neoplastic and anti-neoplastic signals to govern neoplasm growth and neoplasm evolution. Consequently, scrutinizing individual cells at the genome, transcriptome, epigenome, and proteome levels can offer a more comprehensive cellular and molecular level examination [109]. It is apparent that single-cell multi-omics technology holds tremendous significance for investigating neoplasm heterogeneity.

Numerous immune cells within the TME impact tumor development and metastasis. The density, location, and condition of the neoplastic microenvironment assume a crucial role in predicting prognosis and immunotherapy response. To comprehend how neoplasm cells and host tissues affect the immune cell composition in the neoplastic microenvironment, Liu, Y et al. [110] employed single-cell multi-omics technology. They verified the localization of SPP1+ tumor-associated macrophages in tissues using IHC staining and ultimately obtained the necessary experimental conclusions. This study delineates the immune cell landscape across various tissues, defines three immune cell patterns, and enhances our understanding of neoplasm cell characteristics and the impact of tissue distribution on their cancerous phenotypes. All of these findings lay the foundation for advancing single-cell multi-omics technology. In addition to the aforementioned example, the single-cell multi-omics technique also exposes the extensive heterogeneity of stromal cells, encompassing cancer-associated fibroblasts (CAF), infiltrating immune cells, and endothelial cells, during pan-cancer analysis [111].

### 5.2 Reveal the body's immune response

With the rapid advancement of scientific knowledge, single-cell multi-omics technology has emerged as a powerful tool in immunological research, contributing significantly to our understanding of the body's immune response.

In one study, Winkler et al. [112] investigated the role of the gut microbiota in suppressing alphavirus infection through the type I interferon pathway. They employed a combination of microbiome, metabolome, and single-cell transcriptome analysis techniques to uncover the mechanisms involved. In another research effort, Oliveira et al. [113] focused on CD4^+^ Tumor-Infiltrating Lymphocytes (TILs) in human melanoma, specifically examining the differential expression of HLA class II molecules. By utilizing single-cell multi-omics technology, they discovered that CD4^+^ Tregs possess potent immunosuppressive properties. Furthermore, TME was found to be enriched with neoantigen-specific T-Reg cells in HLA class II positive melanoma, suggesting a potential immune escape mechanism. Separately, Keathley et al. [114] utilized an integrated analysis approach combining transcriptome and methylome data. They performed semi-biased clustering which outperformed traditional systematic clustering methods, and subsequently characterized the resulting clusters.

### 5.3 Implications for chemotherapy and cell therapy

Single-cell multi-omics analysis offers simultaneous measurements of genomic, transcriptomic, and proteomic information in a single cell, supporting personalized immunotherapy.

Bassez et al. [115] employed single-cell multi-omics techniques, including single-cell transcriptome, T-cell receptor, and proteome analysis, to elucidate the heterogeneity of breast cancer response to anti-PD1 therapy and explore the mechanism of neoadjuvant chemotherapy in breast cancer. Additionally, Park et al. [116] utilized single-cell multi-omics techniques to demonstrate varied responses to DNA damage from chemotherapy within individual cells of a cancer cell population. The transcriptomic profiles of different cells determined their fate following chemotherapy-induced DNA damage.

## 6 Conclusions, questions, and prospects

Single-cell epigenomics and multi-omics techniques have significantly advanced in various scientific domains, particularly in cancer, stem cells, and immune cells research. These techniques play a crucial role in understanding the body's response to immune checkpoint blockade, chemotherapy, cell therapy, and similar interventions. They enable the scrutiny of the tumor microenvironment at the level of individual cells, uncovering the mechanisms behind patient prognostic variations and drug responses. Moreover, they help comprehend tumor heterogeneity, drug efficacy, and other critical areas of research.

This review focuses on recent advancements in single-cell multi-omics techniques, derived from single-cell epigenomics, and their relevance to tumor biology. It highlights the pivotal role of single-cell epigenomics in oncology, emphasizing its potential to revolutionize cancer research and therapeutics. By providing a comprehensive understanding of tumor heterogeneity and dynamics, these techniques contribute to the identification of new therapeutic targets, patient stratification biomarkers, and personalized treatment strategies, resulting in improved patient outcomes.

In addition, these techniques are instrumental in unraveling the intricate dynamics of immune response and its impact on chemotherapy and cell therapy. They allow researchers to discern the status, function, and subtypes of individual immune cells, as well as their phenotypes and activities, offering invaluable insights into how the immune system responds to tumors. Understanding the interplay between immune cells and their influence on chemotherapy and treatment outcomes emerges as a crucial aspect with profound implications.

Single-cell multi-omics techniques have immense potential in various research fields but are currently limited by complex procedures, limited scope, time-consuming operations, and high costs. However, these techniques can be successfully applied in plants, animals, and medicine, providing valuable insights. To improve these techniques, future advancements are expected to simplify procedures and enhance sensitivity and accuracy, enabling comprehensive analysis of genetic changes in different cell types. Moreover, these techniques could uncover connections between cell types and contribute to the development of spatial omics technologies. Integration with gene editing tools and 3D organoid cultures can expand the applications of single-cell multi-omics and address their limitations. Overall, these techniques are vital in overcoming experimental challenges and facilitating breakthroughs in diverse research endeavors.

### Ethical approval and consent to participate

Not applicable.

## Consent for publication

Not applicable.

## Availability of supporting data

Not applicable.

## Funding

This work was supported partly by The National Natural Science Foundation of China (81972366); Shenzhen High-level Hospital Construction Fund (China); The 10.13039/501100003453Natural Science Foundation of Guangdong, China (2022A1515012606); Shenzhen Project of Science and Technology (Grant No. KCXFZ20211020164005007, and JCYJ20220818101808018).

## CRediT authorship contribution statement

**Anqi Liang:** Investigation, Writing – original draft. **Ying Kong:** Investigation, Writing – review & editing. **Zhihong Chen:** Investigation. **Yishu Qiu:** Writing – review & editing. **Yanhong Wu:** Investigation. **Xiao Zhu:** Conceptualization, Supervision, Writing – review & editing. **Zesong Li:** Funding acquisition.

## Declaration of competing interest

The authors declare that they have no competing interests.

## Data Availability

No data was used for the research described in the article.
